# Distinct spatial transcriptomic patterns of substantia Nigra in Parkinson disease and Parkinsonian subtype of multiple system atrophy

**DOI:** 10.1186/s40478-025-02107-8

**Published:** 2025-09-24

**Authors:** Jung Hwan Shin, Karoliina Eliisa Ruhno, Chaewon Shin, Hyun Je Kim, Soo Jeong Nam, Sun Ju Chung, Ji Hwan Moon, Han-Joon Kim

**Affiliations:** 1 Department of Neurology, Seoul National University Hospital & Seoul National University College of Medicine, 101 Daehak Ro, Jongno-gu, Seoul, 07061 South Korea; 2 Department of Pharmacology, Seoul National University College of Medicine, Seoul, South Korea; 3 Department of Biomedical Sciences, Seoul National University Graduate School, Seoul, South Korea; 4 Department of Neurology, Seoul National University Bundang Hospital, Seongnam-si, Republic of Korea; 5 Department of Pathology, Asan Medical Center, University of Ulsan College of Medicine, Seoul, Republic of Korea; 6 Department of Neurology, Asan Medical Center, University of Ulsan College of Medicine, Seoul, Republic of Korea; 7 Samsung Genome Institute, Samsung Medical Center, Seoul, South Korea

**Keywords:** Spatial transcriptomics, GeoMx, MSA-P, PD, Substantia nigra

## Abstract

To investigate transcriptomic signatures of Parkinson’s disease (PD) and the Parkinsonian subtype of Multiple System Atrophy (MSA-P) in substantia nigra pars compacta (SNpc), we conducted transcriptome analysis using in-situ hybridization on paraffin-embedded SNpc tissues from post-mortem brains. The study included 2 MSA-P patients, 2 PD patients, and 2 healthy controls (HC), with 12 regions of interest (ROIs) selected from the dorsal to ventral and medial to lateral aspects of the SNpc. A total of 72 ROIs from 6 participants were analyzed, and differentially expressed genes (DEGs) were identified by comparing MSA-P, PD and HC groups. The MSA-P group showed 88 upregulated DEGs and 326 downregulated DEGs (adjusted 𝑝<0.05) compared to HC. The downregulated DEGs were significantly enriched in pathways related to ribosomal translation, immune processes, mitochondrial function, and autophagy. Notably, the dorsomedial quadrant was uniquely linked to antigen presentation, while other quadrants showed downregulation of protein synthesis. The PD group exhibited 165 upregulated DEGs and 350 downregulated DEGs (adjusted 𝑝<0.05) compared to HC, with downregulated DEGs associated with ribosomal translation, mitochondrial function, and the ubiquitin-proteasome system. In both MSA-P and PD, the upregulated DEGs were not associated with any pathways or biological process in gene enrichment analysis. In network propagation analysis, amyloid precursor protein was the most significant network hub among DEGs in both MSA-P and PD. Comparing the transcriptomic signatures of SNpc between MSA-P and PD, we found immune/inflammation, mitochondrial function and neural signaling related genes were significantly downregulated in MSA-P compared to PD. Overall, the transcriptomic signature of the SNpc in MSA-P and PD revealed overlapping but distinct features, including alterations in protein synthesis, immune processes, mitochondrial function, and protein degradation systems. Future studies with larger cohorts and functional validation are needed to further elucidate these findings.

**Supplementary Information:**

The online version contains supplementary material available at 10.1186/s40478-025-02107-8.

## Introduction

Parkinson’s disease (PD) and multiple system atrophy (MSA) are both characterized by α-synucleinopathy and present with Parkinsonism as a core clinical feature. PD is marked by presynaptic dopaminergic neuron degeneration associated with a-synuclein aggregation in substantia nigra pars compacta (SNpc) while Parkinsonian subtype of MSA (MSA-P) is characterized by misfolded α-synuclein inclusions predominantly in oligodendrocytes (glial cytoplasmic inclusions), along with involvement of neurons, particularly in the nigrostriatal pathway [50]. MSA-P is associated with three stages of disease severity, starting with early stage MSA, where degeneration is limited to substantia nigra, progressing to more extensive involvement of the putamen, caudate, and globus pallidus in MSA-P [37]. Although in both PD and MSA-P, degeneration extends beyond the nigrostriatal pathway, neurodegeneration in the SNpc play role as a key pathological hallmark. MSA-P can clinically mimic PD, and research has shown that up to 10% of cases initially diagnosed as PD are later confirmed to be MSA, while approximately 7% of MSA cases are misdiagnosed as PD [40]. 

In PD, approximately 30% of dopaminergic neurons are lost at the onset of the disease, while post-mortem studies have shown that dopamine terminal loss in the striatum can reach 80–90%, even though SNpc cell loss may range from only 5–60% [1]. This discrepancy highlights the concept of differential vulnerability within the SNpc, where certain neuronal populations are more susceptible to degeneration than others. Additionally, the degeneration process is thought to involve complex interactions between neurons, microglia, and astrocytes, suggesting a multifaceted degeneration mechanism [9]. These results calls for the single cell based transcriptomic analysis of Substantia nigra in PD or MSA. Furthermore, given the spatial and cellular heterogeneity of degeneration within the SNpc, it is critical to explore how transcriptomic differences contribute to the spatial pattern of pathological processes. In PD, the ventrolateral part of the SNpc is the most vulnerable region to degeneration [12]. However, the spatially distinct transcriptomic signatures within different regions of the SNpc have not been thoroughly investigated in either PD or MSA. Moreover, transcriptomic signatures of SNpc in MSA, particularly MSA-P, has been limited yet [34]. Despite the distinct pathophysiological underpinnings of PD and MSA-P, these disorders often present with overlapping clinical features, particularly in the early stages [54]. Currently, there are no validated biomarkers available that can reliably differentiate PD from MSA-P during life, underscoring the critical need for post-mortem, spatially resolved transcriptomic analyses to uncover disease-specific molecular signatures [26]. 

In this perspective, we performed a whole transcriptome analysis using in-situ hybridization technology on paraffin-embedded substantia nigra tissues from post-mortem human brains of PD, MSA-P and HC.

## Method

### Participants

We retrospectively collected 6 individuals (2 healthy controls, 2 Parkinson disease and 2MSA-P) from Seoul National University Hospital Brain bank and Korea Brain bank Network. 2 HC and 2 MSA-P were from Seoul National University Hospital brain bank and 2 PD cases were collected from Korea brain bank network. All diagnoses were confirmed by post-mortem pathological examination. The local IRB committee approved this study (IB No. 2203-067-1306). We collected the demographics including age, sex, post-mortem interval and pathological diagnosis. We obtained the paraffin imbedded block containing midbrain.

### NanoString GeoMx manual RNA FFPE slide preparation

Nanostring GeoMx experiments were conducted according to manufacturer’s instructions (Manual Slide Preparation MAN-10150-02, NGS DSP Instrument User Manual: MAN-10116-05, Library Preparation & GeoMx NGS Pipeline: MAN-10153-03). 5 μm thick formalin-fixed paraffin-embedded (FFPE) tissue sections were baked for 45 min at 60 °C. The sections were then deparaffinized and rehydrated through 3 × 5 min CitriSolv, 2 × 5 min 100% EtOH, 5 min 95% EtOH and 1-minute 1X PBS wash gradient in staining jars. Target retrieval was then performed in a steamer heated to ~ 99 °C for 10 s in DEPC water and 20 min in 1X Tris-EDTA. This was followed by a 5-minute 1X PBS wash at room temperature. Next, RNA targets were exposed by placing slides in a staining jar containing 0.1 µg/mL of Proteinase K diluted in 1X PBS and incubating them in a 37 °C water bath for 15 min. After this, a 5-minute wash in 1X PBS at room temperature was performed. After RNA target exposure, post-fix preservation was done by washing the slides in 10% NBF for 5 min, then NBF stop buffer for 2 × 5 min, and 1X PBS for 5 min. Then, overnight hybridization at 37 °C was performed in a hybridization chamber by covering each slide with 200 µL of a mixture of Buffer R, Whole Transcriptome Atlas (WTA) RNA detection probes, and DEPC water according to the protocol. The next day, stringent washes were performed in a 37 °C water bath by doing 2 × 25-minute washes in stringent wash solution. Then, 2X SSC washes were done for 2 × 2 min at room temperature. The slides were then covered with 200 µL of Buffer W for blocking in a humidity chamber at room temperature for 30 min, and then with 200 µL of morphology marker mix according to the protocol for 1 h. The morphology markers used were SYTO 13, CD45 (D9M8I, Cell Signaling Technology), GFAP (5C10, Novus), and MBP (2H9, Novus). Finally, 2 × 5-minute 2X SSC washes were performed. After the final wash, the slides were taken to the GeoMx DSP machine.

### Nanostring GeoMx DSP

**Fig. 1:**
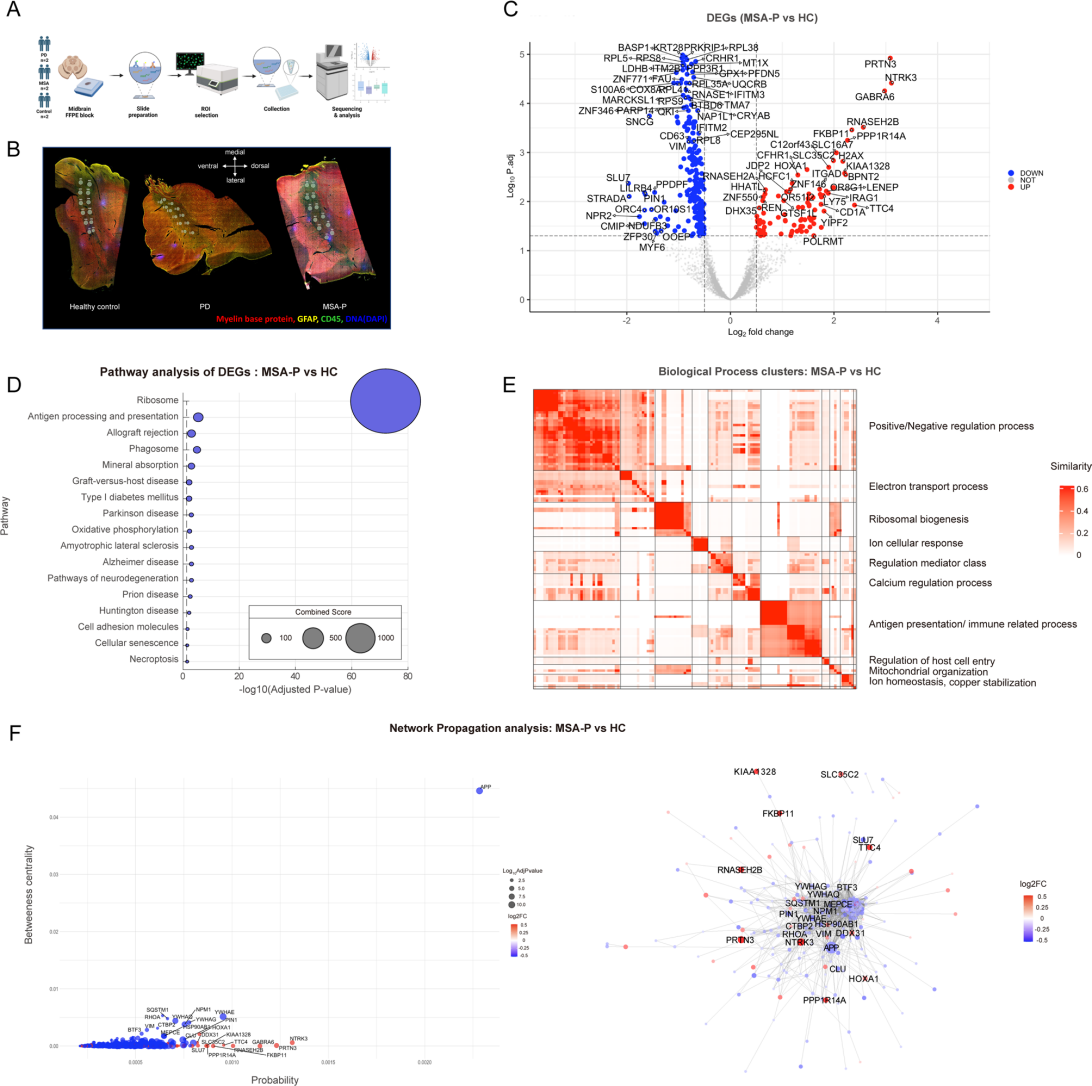
Spatial transcriptomic analysis of substantia nigra in PD, MSA-P, and HC, with a comparison of transcriptomic profiles between MSA-P and HC. (**A**) Schematic flow diagram illustrating the spatiotemporal transcriptomic analysis conducted in this study. (**B**) Representative immunohistological sections from a healthy control, Parkinson’s disease, and MSA-P cases, showing an overlay of four stains (Red: Myelin Basic Protein, Yellow: GFAP, Green: CD45, Blue: DAPI). Regions of interest (ROIs) were selected based on immunohistological patterns to identify cellular ROIs within substantia nigra. (**C**) Volcano plot highlighting significantly upregulated and downregulated differentially expressed genes (DEGs) in MSA-P compared to healthy controls. Pathways are ranked by combined score. (**D**) Pathway analysis using the KEGG 2022 database for downregulated DEGs in MSA-P versus healthy controls shown with adjusted *p*-value and combined scores. (**E**) Gene Ontology (GO) Clusters for biological process. (**F**) The network propagation results visualized using a dot plot (left panel) and a network plot (right panel). The dots and nodes of the genes in those figures are colored in red and blue based on the corresponding genes’ log2 foldchanges (log2FC). Abbreviation; MSA-P: Parkinsonism subtype of multiple system atrophy PD: Parkinson disease, HC: Healthy control

Slides were loaded into the NanoString GeoMx Digital Spatial Profiler (DSP) (NanoString Technologies, Seattle, WA, USA) after being covered with 6 mL of Buffer S. Per slide, we selected 12 regions of interest (ROIs) from substantia nigra pars compacta (SNpc), covering dorsal to ventral and medial to lateral aspects (Fig. 1). ROIs were selected by (J.H.S and K.E.R) based on the topography of SNpc based on 4 morphological marker of MBP, GFAP, CD45 and DAPI. Only ROIs with nuclei count exceeding 90 were selected and the diameter of each ROI was 400 μm. The chosen ROIs were individually illuminated using UV light, allowing the photocleavage of the oligonucleotides from the antibodies bound in the ROIs. These were then collected on a 96-well microwell, which was stored at -20 °C.

**Fig. 2:**
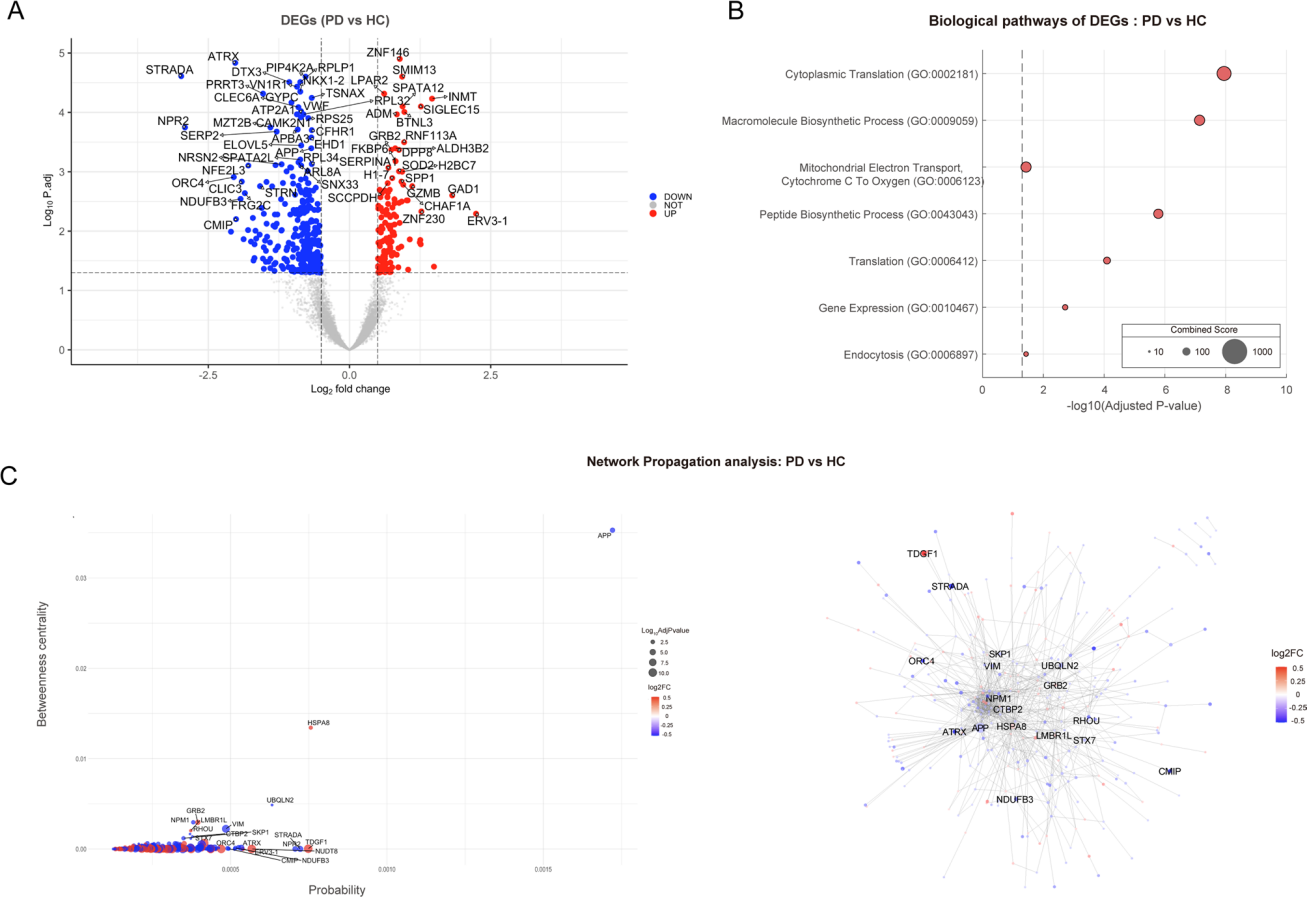
Spatial transcriptomic analysis of substantia nigra comparing PD versus HC. (**A**) The Volcano plot highlighting significantly upregulated and downregulated differentially expressed genes (DEGs) in PD compared to healthy controls. (**B**) Gene Ontology (GO) analysis for downregulated DEGs in comparing PD vs. HC. (**C**) The network propagation results visualized using a dot plot (left panel) and a network plot (right panel). The dots and nodes of the genes in those figures are colored in red and blue based on the corresponding genes’ log2 foldchanges (log2FC). Abbreviation; PD: Parkinson disease, HC: Healthy control

**Fig. 3:**
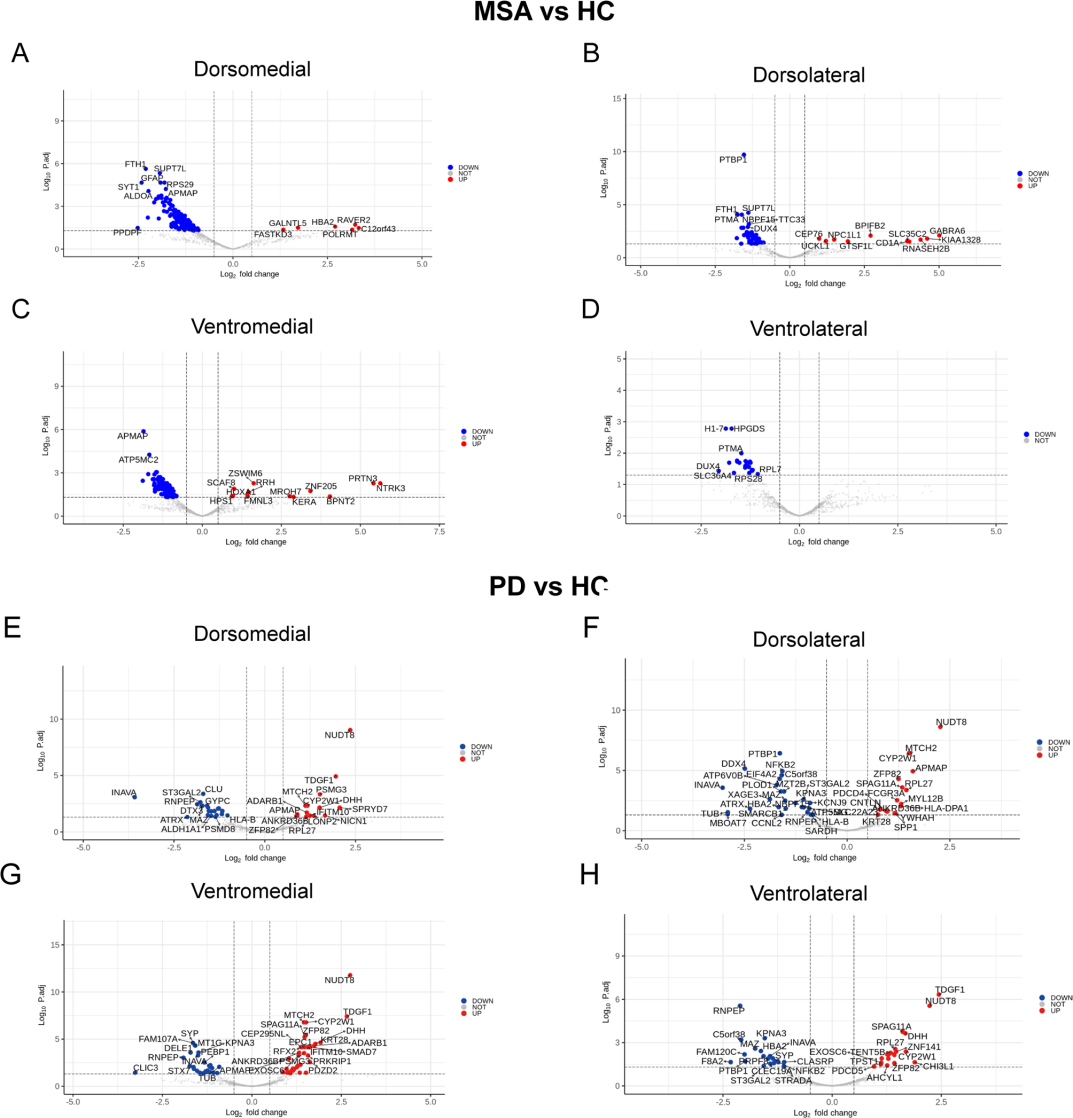
Spatial transcriptomic analysis of quadrants of substantia nigra comparing MSA-P vs. HC. (**A-D**). The Volcano plot highlighting significantly upregulated and downregulated differentially expressed genes (DEGs) in MSA-P compared to healthy controls in dorsomedial (**A**), dorsolateral (**B**), ventromedial (**C**) and ventrolateral (**D**) quadrants. (E-F). The Volcano plot highlighting significantly upregulated and downregulated differentially expressed genes (DEGs) in PD compared to healthy controls in dorsomedial (**E**), dorsolateral (**F**), ventromedial (**G**) and ventrolateral (**H**) quadrants. Abbreviation; MSA-P: Parkinsonism subtype of multiple system atrophy, PD: Parkinson disease, HC: Healthy control

**Fig. 4:**
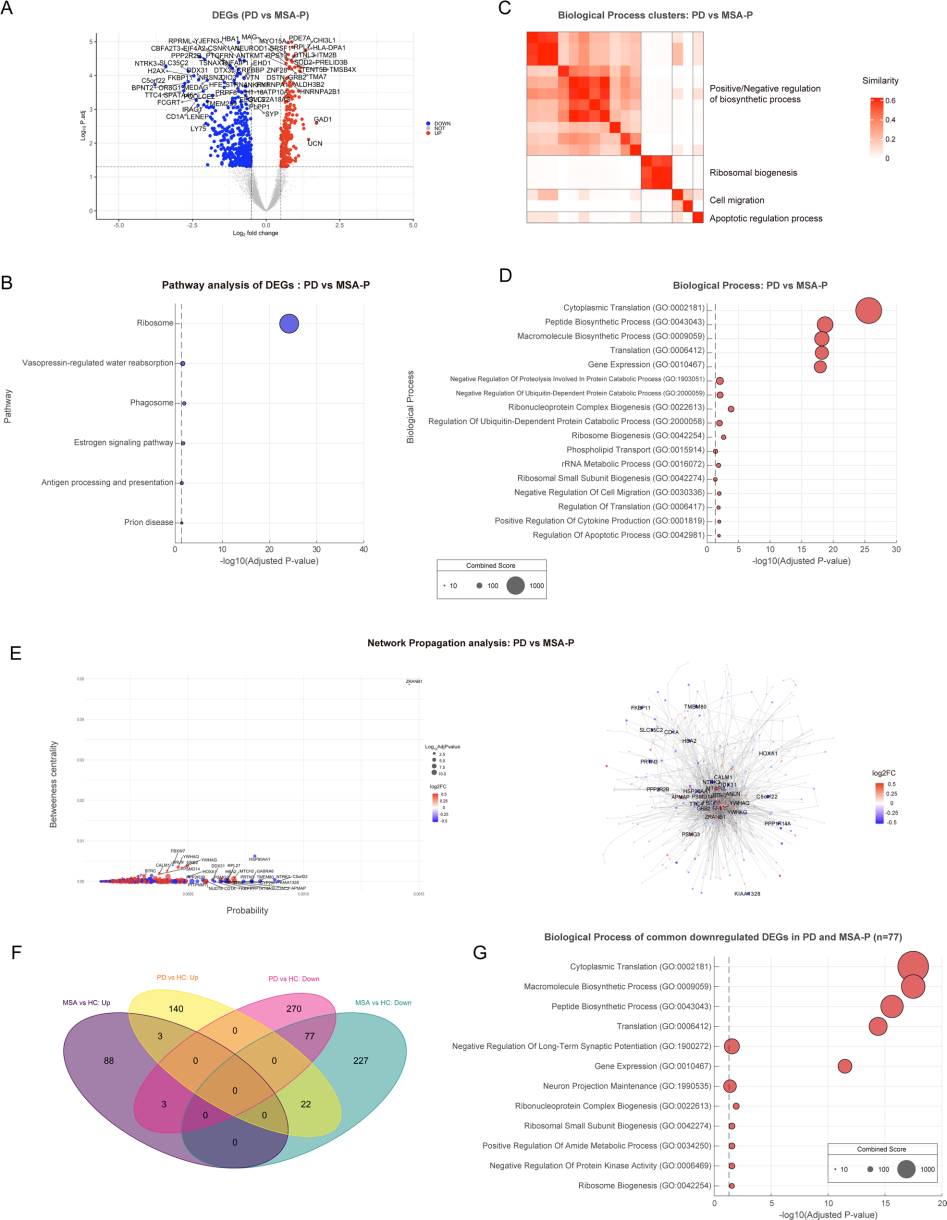
Spatial transcriptomic analysis of substantia nigra comparing MSA-P vs. PD. (**A**) The Volcano plot highlighting significantly upregulated and downregulated differentially expressed genes (DEGs) in MSA-P compared to PD. (**B**) Gene pathway analysis for upregulated DEGs in comparing MSA-P vs. PD. (**C**) Gene Ontology (GO) analysis of upregulated DEGs with each category ranked by combined score. (**D**) Gene Ontology (GO) Clusters for biological process. (**E**) The network propagation results visualized using a dot plot (left panel) and a network plot (right panel). The dots and nodes of the genes in those figures are colored in red and blue based on the corresponding genes’ log2 foldchanges (log2FC). (**F**) Venn diagram showing number of DEGs from comparison of PD and MSA-P with healthy controls. (**G**) Gene Ontology (GO) analysis of the common downregulated genes (*n* = 77) from the comparison of PD and MSA-P with HC, with each category ranked by combined score

### Library generation and pooling

Before library processing, the 96-well plate was thawed, spun down, and dehydrated at 65 °C for 1 h in a thermo-cycler with the lid kept open. Samples were then individually rehydrated with 10 µL of nuclease-free water and prepared for PCR by combining each sample with 2 µL of GeoMx Seq Code PCR Master Mix, 4 µL of primer, and 4 µL of DSP aspirate. After library synthesis, 4 µL of each sample were pooled together and subjected to AMPure cleanup. The pooled sequencing library was then quality controlled using Agilent TapeStation 4150 for each pooled sample group and sequenced on a NovaSeq 6000 instrument (Illumina) using pared-end 2 × 27 base paired reads.

### Data analysis

The raw reads from FASTQ files were processed using NanoString’s GeoMx NGS pipeline version 2.3.3.10 and saved as Digital Count Conversion (DCC) files. Initial quality control was performed using GeoMx DSP Analysis Suite version 2.4.2.2. First, segment and biological probe QC were conducted with preset values. Filtering of the data was not done, and all segments and targets were considered in further analysis. The data were analyzed using R (version 4.4.1) and an in-house pipeline comprising normalization and differential analysis. Normalization was conducted with betweenLaneNormalization [38] and RUVSeq [39]. Differentially expressed genes (DEGs) between the two groups were identified using DESeq2 [27] on the normalized counts. To further investigate the DEGs and pinpoint the key regulators, we conducted network analysis using network propagation on the BioGRID protein-protein interaction (PPI) network [47]. The genes were mapped onto the PPI network, with their corresponding fold change values assigned to the nodes. We applied the random walk with restart (RWR) algorithm to rank the genes based on their network influence. The equation of RWR is as follows:

$$\:{\varvec{p}}^{t+1}=\left(1-r\right){\varvec{W}}^{{\prime\:}}{\varvec{p}}^{t}+r{\varvec{p}}^{0}$$

where $\:{\varvec{p}}^{0}$, $\:{\varvec{p}}^{t}$, and $\:{\varvec{p}}^{t+1}$ are the probability vector of the nodes with their corresponding fold change, the probability vector of the current stage, and the probability vector of the next stage, respectively. $\:{\varvec{W}}^{{\prime\:}}$ is column-normalized adjacency matrix representing the PPI network. $\:r$ is the restart rate.

We also quantified the topological importance of the genes by calculating betweenness centrality on the network. Betweenness centrality quantifies the number of shortest paths between all node pairs that pass through a given node. The equation is shown below:

$$\:BC\left(v\right)=\sum\:_{s\ne\:v\ne\:t\in\:V}\frac{{\sigma\:}_{st}\left(v\right)}{{\sigma\:}_{st}}$$

where $\:V$ is a set of nodes, $\:{\sigma\:}_{st}$ is the total number of shortest paths from node $\:s$ to node $\:v$, and $\:{\sigma\:}_{st}\left(v\right)$ is the number of shortest paths from node $\:s$ to node $\:v$ pass through $\:v$. By utilizing both network propagation result and betweenness centrality, we identified a set of key DEGs. The above data analyses were performed by J.H.M. who were blinded to participant information and ROI selection.

### Gene set enrichment analysis

Gene set enrichment analysis (GSEA) was conducted using Enrichr [21]. For pathway and Gene Ontology (GO) analysis, the KEGG 2021 human database and biological process were utilized to identify associated pathways and terms, respectively [17]. To control for multiple comparisons, adjusted *p*-values were applied, with *p* < 0.05 considered statistically significant. Due to the high redundancy among the enriched terms, we applied simplifyEnrichment [13] to cluster similar terms based on the semantic similarity and improve interpretability. We extracted key biological themes from the clustering results and visualized using heatmaps.

## Result

We enrolled six participants, including two healthy controls (HC), two with Parkinson’s disease (PD), and two with multiple system atrophy with predominant Parkinsonism (MSA-P). Table 1 provides their baseline demographics, pathological features and final diagnoses. The ages at autopsy for the PD, MSA-P, and HC groups were 77 and 77 years, 57 and 71 years, and 74 and 61 years, respectively. The PD group tended to be older. All participants in the MSA-P and HC groups were female, while those in the PD group were male. Both MSA-P and PD cases were pathologically confirmed. In MSA-P, we found that widespread a-synuclein inclusions in both oligodendroglial cells and neurons (Supplementary Fig. 1). The two control cases were diagnosed with primary age-related tauopathy (PART). Participants tested for amyloid and TDP-43 pathologies were negative (Supplementary Table 1). In terms of tau pathology, one PD case exhibited NFT stage 3, and all MSA-P cases were also pathologically diagnosed with PART.

**Table 1:** Demographic and pathological features of the participants

	PD#1	PD#2	MSA-*P*#1	MSA-*P*#2	Control#1	Control#2
Age	77	77	57	71	74	61
Sex	F	F	M	M	M	M
Pathological diagnosis	Parkinson disease	Parkinson disease	Multiple system atrophy with predominant Parkinsonism (MSA-P)	Multiple system atrophy with predominant Parkinsonism (MSA-P)	Primary age related tauopathy	Primary age related tauopathy
Autopsy year	2017	2019	2019	2019	2019	2019
Brain weight (g)	1,100	1,110	1250	1300	1360	1400
Death to perfusion time (hour)	2	1.5	21	16.5	5.5	12.5

Abbreviation; PD: Parkinson disease, MSA-P: Parkinsonism subtype of multiple system atrophy, HC: Healthy control, M: Male, F:Female

### Pathway and biological process analysis: MSA-P versus healthy controls

The MSA-P group exhibited 94 upregulated and 326 downregulated differentially expressed genes (DEGs) compared to healthy controls (Fig. 1C). Pathway analysis of the downregulated DEGs revealed significant associations with protein translation, immune-related processes, and neurodegeneration (Fig. 1D).

For immune-related processes, pathways associated with antigen processing and presentation, phagosome function, and allograft rejection were identified (Fig. 1D). Key involved genes included antigen presentation (HLA-B, HLA-DRA, HLA-A, HLA-DRB1, HLA-E, CD74, B2M), immune cell signaling and activation (FCGR3A, CD40LG), and chaperone-related genes (HSP90AB1, CALR) (adjusted *p* < 0.05).

Regarding neurodegeneration, pathways linked to Parkinson’s disease, oxidative phosphorylation, amyotrophic lateral sclerosis, Alzheimer’s disease, prion disease, and Huntington’s disease were identified (Fig. 1D). The implicated genes included mitochondrial electron transfer system of Complex I (NDUFB3), Complex III (UQCRB, UQCRHL, UQCRQ), Complex IV (COX8A, COX6A1) and Complex V (ATP5MC2). Also, neurodegeneration-related genes (APP, PRNP, PSENEN, RTN3, APOE), calmodulin-related genes (CALM3, CALM2, RPS27A), and autophagy-related genes (SQSTM1, OPTN) were significantly down regulated (adjusted *p* < 0.05; Fig. 1D).

Further analysis of the downregulated DEGs identified 122 gene ontologies related to biological processes (Supplementary Fig. 1). These were categorized into clusters, including ribosomal biosynthesis, antigen processing/immune-related processes, and mitochondrial electron transport (Fig. 1E and Supplementary Table 2). Upregulated DEGs (*n* = 94) in MSA-P did not show any significant associated pathways or biological processes (Supplementary data).

With the network propagation analysis of significant DEGs (both down and upregulated) in MSA-P, we identified genes that were significant in terms of probability and betweenness centrality (Fig. 1F). Notably, the amyloid precursor protein (APP) gene which was downregulated in MSA-P group showed the highest probability and betweenness centrality among all DEGs (Fig. 1F ). From the upregulated genes, stress response pathway (GABRA6, NTRK3, FKBP11) and immune defense mechanisms (RNASEH2B, PRTN3) related genes formed key components of the network in MSA-P. From the downregulated genes, autophagy (SQSTM1), cellular stress response (HSP90AB1, CLU), and protein homeostasis (NPM1, CTBP2, MEPCE, SLU7) related genes were significant hubs within the network.

### Pathway and biological process analysis: PD versus healthy controls

From the whole ROIs, the PD group showed 165 upregulated DEGs and 350 downregulated DEGs (adjusted 𝑝<0.05) compared to healthy controls (Fig. 2A). The pathway analysis revealed significant downregulation in the ribosome pathway (adjusted *p*-value 8.71 × 10-8, combined score 164.36). The ontological analysis in terms of biological process were associated with domains of protein biosynthesis, mitochondrial electron transport and endocytosis pathway with down regulated DEGs (Fig. 2B). The down regulated DEGs included mitochondrial electron transport (COX4I1; COX6A1; COX6C; COX6A2), transcription factor (ATRX; CREB3L4; EIF4A2; FOXG1; GATA6; NFATC1; PTBP1; SOX13), antigen presentation (HLA-B; HLA-DRA), T cell activation and migration (CD40LG, CXCR3, LAG3, TNFAIP1) and B cell function (SPATA2L), genetic stability/DNA repair (RAD51D; SLX4; RNASE1; PTPRD; UBQLN2; DICER1), cell membrane transport/metabolic process (ATP1A3; ATP6V0B; SLC34A2; SLC34A3; SLC44A5; ELOVL5) and cell differentiation (CNTNAP5; HOXB1; HOXB3; CD40LG; TMSB10; CNN3) related genes. However, there were no pathways or biological process associated with upregulated DEGs in PD (Supplementary data).

Through network propagation analysis of significant DEGs in PD vs. HC, we identified genes that were significant in both probability and betweenness centrality. Notably, APP exhibited the highest probability and betweenness centrality among all DEGs in PD (Fig. 2C). Additionally, upregulated genes involved in the stress response pathway, including HSPA8 and NUDT8, emerged as key components of the network. Downregulated genes associated with energy homeostasis (NDUFB3, STRADA, APP) and ubiquitin-proteasome-mediated degradation (UBQLN2, APP, SKP1, NPM1) were significant hub within the network (Fig. 2C).

### Topological feature of transcriptomic signature in MSA-P and PD

In MSA-P, 4 quadrants (dorsomedial, dorsolateral, ventromedial and ventrolateral) showed similar patterns of mainly significant down regulated genes when compared with healthy controls (Fig. 3A-D). The downregulated genes in dorsolateral, ventromedial and ventrolateral ROIs were associated ribosome pathway and protein translation (Supplementary Table 3). However, DEGs from the dorsomedial ROIs were additionally associated with antigen processing and presentation pathways (CD74;HSP90AB1;NFYA; HLA-B; CALR; HLA-DPA1;HLA-E) (Supplementary Fig. 2, Supplementary Table 3). Upregulated genes did not show significant associated pathway in every quadrants in MSA-P (adjusted *p* value > 0.05). In the quadrant analysis of PD, there were no significant pathways or ontological processes were identified in any quadrant (Fig. 3E-H).

### Pathway and biological process analysis: PD vs. MSA-P

From the total ROIs, the PD group showed 323 upregulated DEGs and 471 downregulated DEGs (adjusted 𝑝<0.05) compared to MSA-P. (Fig. 4A). The upregulated DEGs (PD > MSA-P) in PD showed association with ribosome, phagosome, estrogen signaling pathway, antigen processing and presentation and prion disease in pathway analysis (Fig. 4B). Pathways regarding Parkinson disease, Alzheimer’s disease and necroptosis showed trend of upregulated DEGs (adjusted *p*-value < 0.1). The related genes included immune and inflammation related genes including antigen presentation (CD74; HLA-DRB1; HLA-DPA1), chaperone-related (HSP90AB1; NFYA), immune cell signaling (FCGR3A; FCER1G) and complement system (C1QB; C1QC), mitochondria/electron transfer related genes (ATP5PD; PSMD14; UQCRC1; ATP5MC2; CYBB; VDAC1) and neural signaling pathway (CREB3; HSP90AB1; PIK3R1; GRB2; ADCY5; CTSD; CALM1; CALM2; CALM1; CALM2; PIK3R1; ADCY5; GRIN2A; WNT10A). The significantly associated biological processes with downregulated DEGs mainly involved protein synthetic process, cell migration and apoptotic regulation process (Fig. 4C-D, Supplementary Table 4). However, there were no pathways or biological process associated with downregulated genes (MSA-*P* > PD) in PD (Supplementary data). Through network propagation analysis of significant DEGs in PD vs. MSA-P, we identified upregulated genes in PD (PD > MSA-P) that are associated with mitochondrial function (NUDT8, MTCH2) and cellular stress response (YWHAQ, YWHAG, CALM1) (Fig. 4E). Among the downregulated genes in PD (MSA-*P* > PD), key components included those involved in stress adaptation (HSP90AA1, GABRA6, NTRK3, DDX31), signal transduction (HSP90AA1, NTRK3, BTRC, PITPNM1, PPP2R2B), and ribosomal biogenesis (RPL27, DDX31) (Fig. 4E). Among the significant DEGs in MSA-P and PD compared to HC, only 77 downregulated DEGs (22.2%, 25.3% in MSA-P and PD, respectively) and 3 upregulated DEGs (3.3%, 2.1% in MSA-P and PD, respectively) were shared (Fig. 4F, Supplementary Table 5). Pathway analysis of the common downregulated DEGs (*n* = 77) revealed an association with the ribosome (combined score: 1794.82, adjusted *p* = 2.78 × 10⁻¹⁸). Additionally, biological process analysis of the common DEGs indicated an association with protein translation (Fig. 4G).

## Discussion

This study aimed to characterize the transcriptomic signatures of the SNpc in MSA-P and PD which revealed distinct transcriptomic signatures associated with various biological pathways, including protein synthesis, mitochondrial function, and immune-related processes. These findings may reflect long-term transcriptomic adaptations to neurodegeneration in the SNpc in MSA-P and PD.

### Transcriptomic signature of MSA-P and PD: down regulation of ribosomal biosynthesis

Notably, among the downregulated pathways, ribosomal protein synthesis genes showed the significant association in our data, highlighting a potential role of impaired protein translation in MSA-P and PD. From the neurodegenerative perspective, dysregulation in protein synthesis has been known to impaired in Alzheimer’s disease and Parkinson disease, especially from the early disease course [6, 7, 10, 31]. In Parkinsonian mouse and cell model, the most prominent biological processes interacting with pathological α-synuclein included RNA processing and translation initiation [18]. In Parkinson disease, LRRK2, a major genetic risk factor for PD, is known to negatively regulate protein synthesis via miRNA [11]. Moreover, altered protein translation has been reported in substantia nigra of post-mortem human brains, with a notable decrease in Nucleophosmin (NPM1) mRNA expression in the SNpc [10]. NPM1 was consistently downregulated in our data from both PD and MSA SNpc, and emerged as a key gene in the network propagation analysis (Figs. 1F and 2C). In MSA, the paraffin embedded brain samples from rostral pons in MSA patient showed decreased protein modification, protein synthesis/transport [22] which was also reported from whole blood [35] and miRNA analysis [51]. Furthermore, altered protein synthesis was reported as differential transcriptional profile in transgenic mouse model of MSA [43]. However, the altered protein translation transcriptomic profile in the post-mortem SNpc from MSA-P was described for the first time in this study [34]. Overall, we showed profound down regulated DEGs in substantia nigra in both MSA-P and PD human brain which supports the hypothesis of impaired ribosomal dysfunction and reduced protein synthesis in substantia nigra in both Parkinsonian syndromes.

### Transcriptomic signature of MSA-P and PD: immune related process

In pathway analysis, MSA-P showed significant downregulated DEGs regarding immune related process involving antigen processing and presentation, inflammation and chaperone related genes in SNpc (Fig. 1D, E). Numerous previous studies accumulated evidence of inflammation and an enhanced immune response in both the brain and peripheral tissues, including blood in the pathogenesis of MSA and PD.

In the previous transcriptomic analysis using post-mortem brain tissue, there are conflicting results showing upregulation or downregulation of immune response pathways in PD and MSA. In PD, several studies showed upregulation of inflammatory/immune related pathway in the post-mortem tissue including putamen [25], frontal gyrus [2], substantia nigra [20, 28]. Especially, the activation of microglial in neuroinflammation has been highlighted in single cell sequencing study of SNpc in PD [16, 24, 28]. Also in MSA, the activation of immune process including microglial antigen presentation and elevated pro-inflammatory cytokines were reported in prefrontal cortex [41] and pons [22].

On the other hand, transcriptomic analysis study using post-mortem frontal gyrus in PD showed that immune response pathways were significantly upregulated in early disease course but down-regulated at most advanced stage of the disease [2]. Also in MSA, post-mortem study using frozen tissue of both grey matter and white matter in MSA showed down regulation of HLA class compared to healthy controls [32]. 

Thus, in the advanced stage, the immune-related pathways in the SNpc may be associated with the downregulation of immune-related genes, potentially reflecting a compensatory adaptation to prolonged disease progression. Given that the post-mortem stage likely represents a more advanced disease state than observed in living patients, this notion is partially supported by blood-based studies from living MSA patients, which consistently show enrichment of immune and inflammation-related differentially expressed genes (DEGs) and proinflammatory cytokines [19] in MSA [35, 41]. Notably, our findings are derived from the SNpc, a region for which transcriptomic analysis in post-mortem MSA brains has not yet been reported [34]. Thus further validation is needed. Nevertheless, the significant downregulation of these immune-related genes in MSA-P provides insight into the longitudinal dynamics of inflammation in disease pathogenesis. In contrast to MSA-P, the alteration of immune or inflammatory processes was not significantly represented in the pathway analysis of our PD dataset. The comparison of PD versus MSA-P in immune related process will be discussed in the later section.

### Transcriptomic signature of MSA-P and PD: neurodegenerative process

The involvement of mitochondrial function, autophagy and ubiquitin-proteasome system has been known as the key pathophysiological pathway of neurodegeneration underlying MSA and PD [5]. In mitochondrial electron transport pathways, we found the significant downregulation of pathways in both MSA-P and PD in substantia nigra. Especially, DEGs for multiple complexes in mitochondrial electron transport pathway (I, III, IV and V) were impaired in SNpc of MSA-P. This result is consistent with the previous post-mortem tissue analysis in pons [22] of MSA-P showing decreased expression of Complex I ~ IV units in the electron transfer chain in the mitochondria. In the MSA-P, SQSTM1 which is related with autophagy was significantly downregulated and also was a significant hub in the network transcriptomic analysis in our data. Previous studies have also shown alteration of the autophagy in MSA based on micro RNA studies from post mortem brain [23, 49, 51]. Furthermore, the polymorphism of SQSTM1 is associated with increased genetic risk of MSA [48]. 

The DEGs for ubiquitin-proteasome system (UPS) mediated degradation (UBQLN2, SKP1, NPM1) were significantly downregulated in PD but was not evident in MSA-P in our data [45]. This result is in line with the previous report showing impaired UPS in post-mortem SN tissue of PD patients [3, 30]. Regarding the stress response pathway, the altered genes related with stress pathway were both involved in MSA-P (GABRA6, NTRK3, FKBP11 HSP90AB1, CLU) and PD (HSPA8 and NUDT8) which were also the significant hub in the network propagation analysis.

Notably, APP was the most significant hub gene and was significantly downregulated in the SNpc of both PD and MSA-P compared to HC. APP is primarily known for its role in the generation of amyloid-beta peptides associated with Alzheimer’s disease (AD). Beyond its role in AD, APP plays a crucial role in neuronal development and synaptic plasticity [57]. It is involved in the modulation of essential metal ions, such as iron and copper, which are vital for oxygen transport, mitochondrial respiration, and DNA synthesis. Dysregulation of these metal ions has been implicated as a key pathological mechanism underlying dopaminergic neuronal degeneration [53]. Additionally, APP contributes to several neuroprotective functions through its soluble cleavage products, particularly APPsα, which supports synaptic integrity [8]. APP is also involved in calcium regulation and synaptic stability, with its deficiency being linked to impaired long-term potentiation (LTP) and disrupted GABAergic signaling in various neuronal contexts [14]. Specifically, APP’s modulation of Cav1.2 L-type calcium channels may influence GABAergic synaptic strength, further underscoring its potential role in neurodegenerative processes [56]. In contrast to the amyloidogenic process in AD, where APP is upregulated [29], its downregulation and potential loss of function may contribute to the pathogenesis of PD and MSA-P. A previous transcriptomic study identified a functional network associated with APP in MSA-C [36]. Genetic variants in APP have been associated with modifying the clinical phenotype of PD and some of the variants were frequently found in PD than AD group [44]. Notably, the co-pathology of amyloid was weak in all participants in this study (Supplementary Table 1). These results suggest that the observed APP downregulation may reflect mechanisms independent of amyloid-driven neurodegeneration, potentially implicating APP in synuclein-related disease pathways affecting SNpc. Further investigation is needed to clarify the functional implications of APP downregulation in MSA-P and PD.

### Transcriptomic difference between MSA-P and PD

Comparison of DEGs in the SNpc between PD and MSA-P revealed downregulation of protein synthesis, and antigen presentation pathways in MSA-P, as evidenced by pathway analysis. In terms of biological processes, MSA-P exhibited downregulation of genes associated with immune and inflammatory responses, mitochondrial and electron transfer functions, and neural signaling pathways compared to PD. Thus, together with the results from comparisons between HC and both PD and MSA-P, the downregulation of DEGs related to protein translation, mitochondrial function, immune-related and neural signaling pathways was more pronounced in MSA-P than in PD. Especially for the inflammation and immune related process, previous studies showed more prominent involvement of these pathway in MSA compared to PD based on altered CSF inflammatory proteins and cytokines [42, 46], high serum monocyte-to-high-density lipoprotein ratio (MHR) [15]. As immune-related pathways were downregulated in MSA-P compared to HC, the further downregulation in MSA-P compared to PD suggests a long-term adaptation to the more pronounced immune and inflammatory processes in MSA-P. However, this does not necessarily imply a lack of contribution from immune-related pathways in PD, as several DEGs related to immune processes (CD40LG, CXCR3, LAG3, IFIH1, HLA-B, HLA-DRA, SPATA2L, TNFAIP1), including those involved in antigen presentation and T cell and B cell immune functions, were downregulated in PD. Nevertheless, these DEGs did not show significant associations with pathway based on gene enrichment analysis, suggesting that the immune process alterations in PD were relatively mild compared to MSA-P in SNpc. In the network propagation analysis, downregulated genes in MSA-P were associated with mitochondrial function and stress response, while upregulated genes related to stress adaptation and signal transduction emerged as significant hubs within the network. Notably, the significant DEGs (both up- and downregulated) showed minimal overlap between PD and MSA-P when compared to HC (15.5% and 19.0% in PD and MSA-P, respectively), suggesting distinct transcriptional signatures in the SNpc of MSA-P and HC [35]. However, pathway and biological process analyses revealed that the common downregulated DEGs were associated with ribosomal function and protein translation (Fig. 4G). This further supports the hypothesis of impaired ribosomal function and reduced protein synthesis in the SNpc in both MSA-P and PD, indicating shared biological processes.

### Transcriptomic signature of MSA-P and PD: topographical organization

In our study, there was the regional specificity of its transcriptomic alterations within the SNpc in MSA-P. Specifically, the dorsomedial quadrant exhibited a significant enrichment of antigen presentation-related pathways compared to other quadrants mainly involving protein synthetic pathway (Supplementary Table 3). There has been no direct evidence of topographical degeneration pattern of SNpc in MSA-P. However, the dopamine binding pattern observed with FP-CIT PET in MSA-P showed a significant decrease in the ventral striatum, to which the dorsomedial SNpc preferentially projects [4], compared to both HC and age, sex, and disease duration matched PD [33]. However, the susceptibility of dorsomedial SN require cautious interpretation as the dopamine transporter binding is much lower in the posterior putamen than ventral striatum of MSA-P [33]. Furthermore, the degree of neurodegeneration within SNpc was not quantitatively assessed in our dataset. Nevertheless, future studies exploring the topographic susceptibility of SN degeneration in MSA is warranted. On the other hand, PD did not show pronounced quadrant-specific transcriptional differences which is in contrast to the previous study showing susceptible transcriptional clusters localized in ventral part of SN [16, 52, 55]. 

Our study has several limitations. The small sample size limits generalizability. Variability in post-mortem tissue processing, including fixation times, may introduce inconsistencies. Additionally, the ROI-based spatial transcriptomic analysis used in this study could not resolve cell-type-specific transcriptomic profiles, limiting the identification of neuronal- and glial-specific transcriptomic features. Furthermore, trajectory-based analyses, such as pseudotime resolution analysis, could not be performed due to the lack of single-cell resolution, highlighting the need for future studies using single-cell spatial transcriptomics. The decreased expression of APP could not be validated by immunohistochemistry due to the unavailability of additional midbrain tissue sections from the participants. The study only included SNpc thus may not be generalized to brainstem, cerebellum and striatum which is also the main regions that are affected in MSA-P.

In conclusion, our study offers novel insights into the transcriptomic landscape of MSA-P and PD, highlighting key differences in protein synthesis, mitochondrial function, immune processes, stress response, autophagy and ubiquitin-proteasome system. MSA-P and PD shared common pathways of downregulated ribosomal protein synthesis in SNpc. However, MSA-P showed more pronounced down regulation of immune and inflammation related process, mitochondrial/electron transfer functional pathways and autophagy compared to PD. The prominent role of APP as a network hub in both PD and MSA-P suggested involvement in synucleinopathy pathogenesis beyond amyloid-related mechanisms. Future studies with larger cohorts and functional validation are essential to further clarify these findings.

## Supplementary Information

Below is the link to the electronic supplementary material.

Supplementary Material 1

Supplementary Material 2

## Data Availability

The raw (normalized expression counts) and processed data (DEGs) are available in the public data repository (10.6084/m9.figshare.29323688).
