# Correction: Distinct spatial transcriptomic patterns of substantia Nigra in Parkinson disease and Parkinsonian subtype of multiple system atrophy

**DOI:** 10.1186/s40478-025-02157-y

**Published:** 2025-11-04

**Authors:** Jung Hwan Shin, Karoliina Eliisa Ruhno, Chaewon Shin, Hyun Je Kim, Soo Jeong Nam, Sun Ju Chung, Ji Hwan Moon, Han-Joon Kim

**Affiliations:** 1 Department of Neurology, Seoul National University Hospital & Seoul National University College of Medicine, 101 Daehak Ro, Jongno-Gu, Seoul, 07061 South Korea; 2 Department of Pharmacology, Seoul National University College of Medicine, Seoul, South Korea; 3 Department of Biomedical Sciences, Seoul National University Graduate School, Seoul, South Korea; 4 Department of Neurology, Seoul National University Bundang Hospital, Seongnam-si, Republic of Korea; 5 Department of Pathology, Asan Medical Center, University of Ulsan College of Medicine, Seoul, Republic of Korea; 6 Department of Neurology, Asan Medical Center, University of Ulsan College of Medicine, Seoul, Republic of Korea; 7 Samsung Genome Institute, Samsung Medical Center, Seoul, South Korea

**Correction to: Acta Neuropathologica Communications (2025) 13:193** 10.1186/s40478-025-02107-8

In this article [1], the affiliation details for the authors’ Soo Jeong Nam and Sun Ju Chung had been interchanged and should have been

Soo Jeong Nam

Department of Pathology, Asan Medical Center, University of Ulsan College of Medicine, Seoul, Republic of Korea

Sun Ju Chung

Department of Neurology, Asan Medical Center, University of Ulsan College of Medicine, Seoul, Republic of Korea

The original article has been corrected.
