# Supplementary material for: Distinct spatial transcriptomic patterns of substantia Nigra in Parkinson disease and Parkinsonian subtype of multiple system atrophy
Source: Acta Neuropathol Commun. 2025 Sep 24;13:193. doi: 10.1186/s40478-025-02107-8 (PMC12462216; doi:10.1186/s40478-025-02107-8)
Supplement: Supplementary file 1 — Supplementary Material 1 [file 40478_2025_2107_MOESM1_ESM.docx]

Supplementary Figure 1. Immunohistochemistry of MSA-P patients


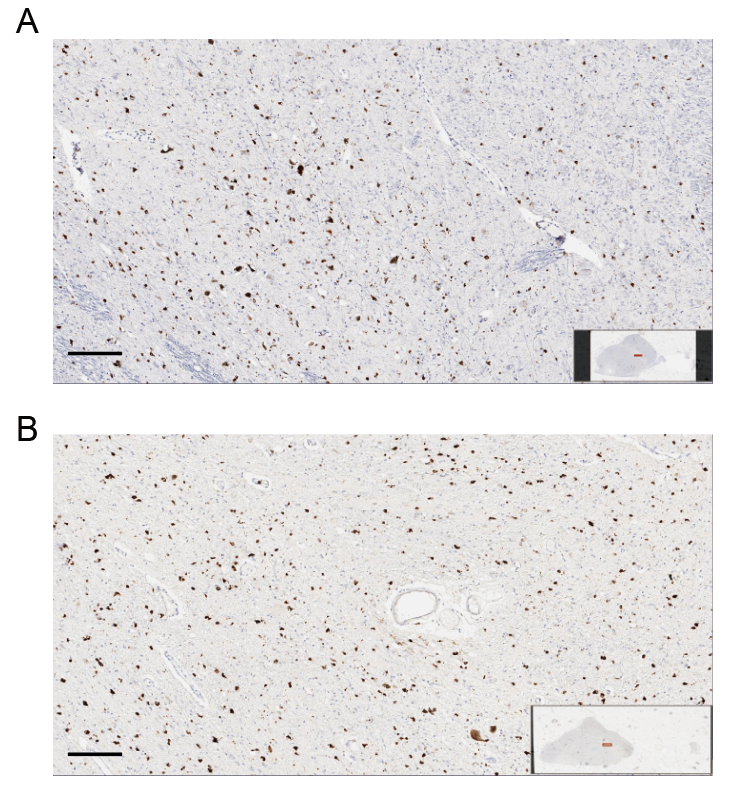


A–B. α-Synuclein immunohistochemistry of the SNpc in MSA-P patients (A: MSA-P #1, B: MSA-P #2) demonstrating widespread and abundant neuronal and oligodendroglial cytoplasmic inclusions.

Scale bar: 200 μm; inset indicates the relative position (red box) of the imaging window within the midbrain section.

Supplementary Figure 2. Biological process associated with downregulated DEGs in MSA-P versus healthy control


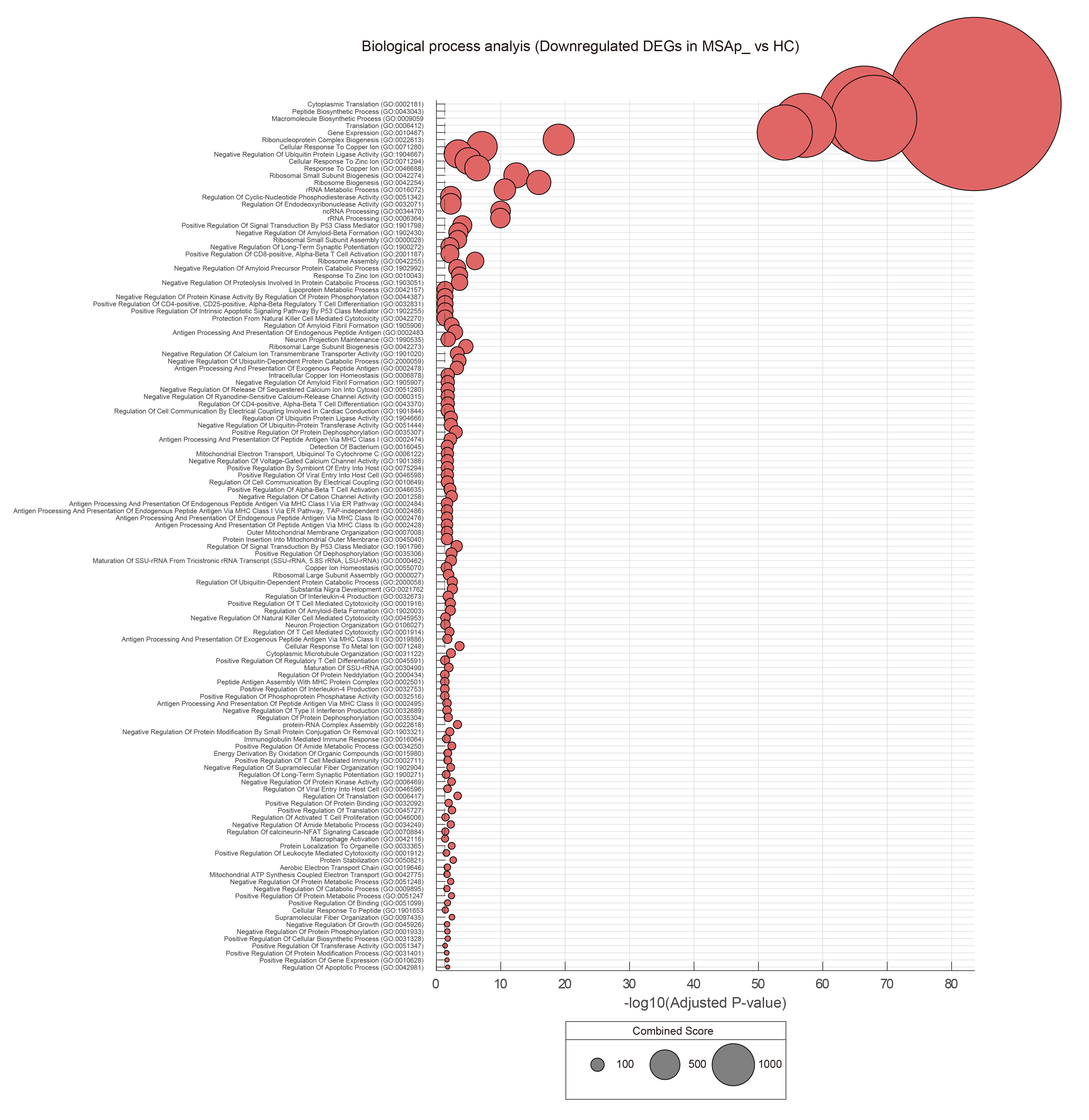


Supplementary Figure 3. Pathway and Biological process associated with downregulated DEGs in dorsomedial quadrant in MSA-P versus healthy control


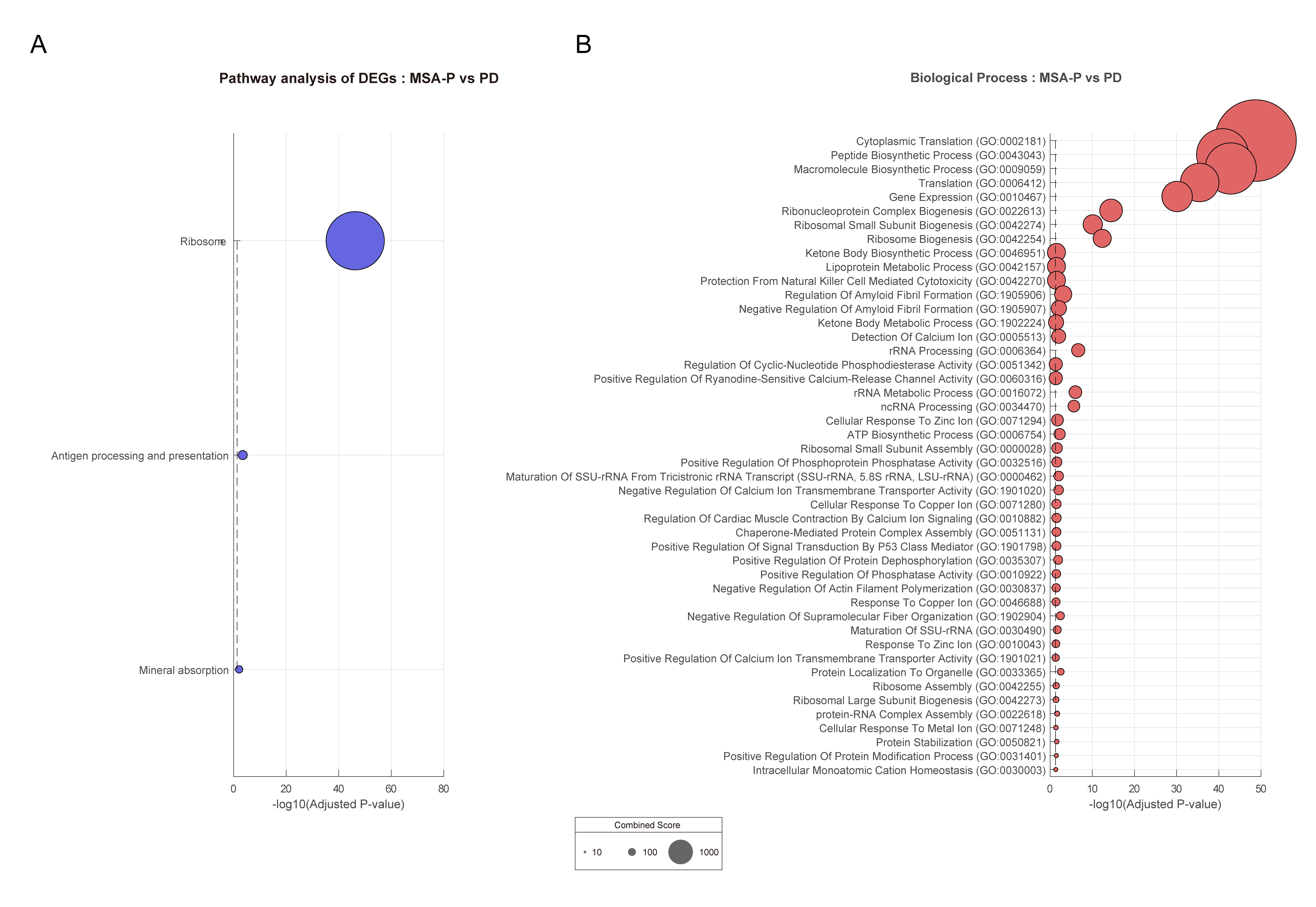


A. Pathway analysis using the KEGG 2022 database for upregulated DEGs in MSA-P versus PD as shown with adjusted pvalue and combined scores. B. Gene Ontology (GO) for biological process as shown with adjusted pvalue and combined scores

|  | PD#1 | PD#2 | MSA-P#1 | MSA-P#2 | Control#1 | Control#2 |
| --- | --- | --- | --- | --- | --- | --- |
| Pathological stage (Synuclein) | Braak stage IV | Braak stage IV | Widespread of oligodendroglial cytoplasmic inclusions | Widespread of oligodendroglial cytoplasmic inclusions | Negative | Negative |
| Pathological stage  (Amyloid) | N.A. | Thal phase 1 | Thal phase 0 | Thal phase 1 | Thal phase 0 | Thal phase 0 |
| Pathological stage (Tau) | N.A. | Braak NFT stage III | Primary age related tauopathy | Primary age related tauopathy | Primary age related tauopathy | Primary age related tauopathy |
| Pathological stage (TDP-43) | N.A. | Negative | Negative | Negative | Negative | Negative |

Supplementary Table 1. Co-pathologies in participants

Supplementary Table 2. Gene ontology (Biological Process) clusters for downregulated genes in MSA-P vs HC

|  | id | term | cluster |
| --- | --- | --- | --- |
| 1 | GO:0002181 | cytoplasmic translation | 1 |
| 2 | GO:0043043 | peptide biosynthetic process | 1 |
| 3 | GO:0006412 | translation | 1 |
| 4 | GO:1903051 | negative regulation of proteolysis involved in cellular protein catabolic process | 1 |
| 5 | GO:2000059 | negative regulation of ubiquitin-dependent protein catabolic process | 1 |
| 6 | GO:1902430 | negative regulation of amyloid-beta formation | 1 |
| 7 | GO:1904667 | negative regulation of ubiquitin protein ligase activity | 1 |
| 8 | GO:0006417 | regulation of translation | 1 |
| 9 | GO:1902992 | negative regulation of amyloid precursor protein catabolic process | 1 |
| 10 | GO:0035307 | positive regulation of protein dephosphorylation | 1 |
| 11 | GO:2000058 | regulation of ubiquitin-dependent protein catabolic process | 1 |
| 12 | GO:0045727 | positive regulation of translation | 1 |
| 13 | GO:0034250 | positive regulation of cellular amide metabolic process | 1 |
| 14 | GO:1905906 | regulation of amyloid fibril formation | 1 |
| 15 | GO:0051247 | positive regulation of protein metabolic process | 1 |
| 16 | GO:0006469 | negative regulation of protein kinase activity | 1 |
| 17 | GO:0035306 | positive regulation of dephosphorylation | 1 |
| 18 | GO:0051444 | negative regulation of ubiquitin-protein transferase activity | 1 |
| 19 | GO:0034249 | negative regulation of cellular amide metabolic process | 1 |
| 20 | GO:0051248 | negative regulation of protein metabolic process | 1 |
| 21 | GO:1902003 | regulation of amyloid-beta formation | 1 |
| 22 | GO:1903321 | negative regulation of protein modification by small protein conjugation or removal | 1 |
| 23 | GO:0035304 | regulation of protein dephosphorylation | 1 |
| 24 | GO:0031328 | positive regulation of cellular biosynthetic process | 1 |
| 25 | GO:1905907 | negative regulation of amyloid fibril formation | 1 |
| 26 | GO:0001933 | negative regulation of protein phosphorylation | 1 |
| 27 | GO:0010628 | positive regulation of gene expression | 1 |
| 28 | GO:0032689 | negative regulation of interferon-gamma production | 1 |
| 29 | GO:0009895 | negative regulation of catabolic process | 1 |
| 30 | GO:0031401 | positive regulation of protein modification process | 1 |
| 31 | GO:0044387 | negative regulation of protein kinase activity by regulation of protein phosphorylation | 1 |
| 32 | GO:2000434 | regulation of protein neddylation | 1 |
| 33 | GO:0032516 | positive regulation of phosphoprotein phosphatase activity | 1 |
| 34 | GO:0009059 | macromolecule biosynthetic process | 2 |
| 35 | GO:0010467 | gene expression | 2 |
| 36 | GO:0016072 | rRNA metabolic process | 2 |
| 37 | GO:0034470 | ncRNA processing | 2 |
| 38 | GO:0021762 | substantia nigra development | 2 |
| 39 | GO:0032071 | regulation of endodeoxyribonuclease activity | 2 |
| 40 | GO:0032673 | regulation of interleukin-4 production | 2 |
| 41 | GO:0015980 | energy derivation by oxidation of organic compounds | 2 |
| 42 | GO:0019646 | aerobic electron transport chain | 2 |
| 43 | GO:0006122 | mitochondrial electron transport, ubiquinol to cytochrome c | 2 |
| 44 | GO:0042775 | mitochondrial ATP synthesis coupled electron transport | 2 |
| 45 | GO:0042157 | lipoprotein metabolic process | 2 |
| 46 | GO:0032753 | positive regulation of interleukin-4 production | 2 |
| 47 | GO:0022613 | ribonucleoprotein complex biogenesis | 3 |
| 48 | GO:0042254 | ribosome biogenesis | 3 |
| 49 | GO:0042274 | ribosomal small subunit biogenesis | 3 |
| 50 | GO:0006364 | rRNA processing | 3 |
| 51 | GO:0042255 | ribosome assembly | 3 |
| 52 | GO:0042273 | ribosomal large subunit biogenesis | 3 |
| 53 | GO:0000028 | ribosomal small subunit assembly | 3 |
| 54 | GO:0022618 | ribonucleoprotein complex assembly | 3 |
| 55 | GO:0097435 | supramolecular fiber organization | 3 |
| 56 | GO:0000462 | maturation of SSU-rRNA from tricistronic rRNA transcript (SSU-rRNA, 5.8S rRNA, LSU-rRNA) | 3 |
| 57 | GO:0031122 | cytoplasmic microtubule organization | 3 |
| 58 | GO:0030490 | maturation of SSU-rRNA | 3 |
| 59 | GO:0000027 | ribosomal large subunit assembly | 3 |
| 60 | GO:0106027 | neuron projection organization | 3 |
| 61 | GO:0071280 | cellular response to copper ion | 4 |
| 62 | GO:0046688 | response to copper ion | 4 |
| 63 | GO:0071294 | cellular response to zinc ion | 4 |
| 64 | GO:0071248 | cellular response to metal ion | 4 |
| 65 | GO:0010043 | response to zinc ion | 4 |
| 66 | GO:1901653 | cellular response to peptide | 4 |
| 67 | GO:1901798 | positive regulation of signal transduction by p53 class mediator | 5 |
| 68 | GO:1901796 | regulation of signal transduction by p53 class mediator | 5 |
| 69 | GO:1900272 | negative regulation of long-term synaptic potentiation | 5 |
| 70 | GO:0042981 | regulation of apoptotic process | 5 |
| 71 | GO:1901844 | regulation of cell communication by electrical coupling involved in cardiac conduction | 5 |
| 72 | GO:0010649 | regulation of cell communication by electrical coupling | 5 |
| 73 | GO:1900271 | regulation of long-term synaptic potentiation | 5 |
| 74 | GO:0070884 | regulation of calcineurin-NFAT signaling cascade | 5 |
| 75 | GO:1902255 | positive regulation of intrinsic apoptotic signaling pathway by p53 class mediator | 5 |
| 76 | GO:1901020 | negative regulation of calcium ion transmembrane transporter activity | 6 |
| 77 | GO:2001258 | negative regulation of cation channel activity | 6 |
| 78 | GO:0033365 | protein localization to organelle | 6 |
| 79 | GO:1904666 | regulation of ubiquitin protein ligase activity | 6 |
| 80 | GO:0051342 | regulation of cyclic-nucleotide phosphodiesterase activity | 6 |
| 81 | GO:0032092 | positive regulation of protein binding | 6 |
| 82 | GO:0051280 | negative regulation of release of sequestered calcium ion into cytosol | 6 |
| 83 | GO:0060315 | negative regulation of ryanodine-sensitive calcium-release channel activity | 6 |
| 84 | GO:0051099 | positive regulation of binding | 6 |
| 85 | GO:1901386 | negative regulation of voltage-gated calcium channel activity | 6 |
| 86 | GO:0051347 | positive regulation of transferase activity | 6 |
| 87 | GO:0002478 | antigen processing and presentation of exogenous peptide antigen | 7 |
| 88 | GO:0002483 | antigen processing and presentation of endogenous peptide antigen | 7 |
| 89 | GO:0002474 | antigen processing and presentation of peptide antigen via MHC class I | 7 |
| 90 | GO:0001916 | positive regulation of T cell mediated cytotoxicity | 7 |
| 91 | GO:0046635 | positive regulation of alpha-beta T cell activation | 7 |
| 92 | GO:2001187 | positive regulation of CD8-positive, alpha-beta T cell activation | 7 |
| 93 | GO:0001914 | regulation of T cell mediated cytotoxicity | 7 |
| 94 | GO:0043370 | regulation of CD4-positive, alpha-beta T cell differentiation | 7 |
| 95 | GO:0002711 | positive regulation of T cell mediated immunity | 7 |
| 96 | GO:0019886 | antigen processing and presentation of exogenous peptide antigen via MHC class II | 7 |
| 97 | GO:0002495 | antigen processing and presentation of peptide antigen via MHC class II | 7 |
| 98 | GO:0002484 | antigen processing and presentation of endogenous peptide antigen via MHC class I via ER pathway | 7 |
| 99 | GO:0002486 | antigen processing and presentation of endogenous peptide antigen via MHC class I via ER pathway, TAP-independent | 7 |
| 100 | GO:0002476 | antigen processing and presentation of endogenous peptide antigen via MHC class Ib | 7 |
| 101 | GO:0002428 | antigen processing and presentation of peptide antigen via MHC class Ib | 7 |
| 102 | GO:0016064 | immunoglobulin mediated immune response | 7 |
| 103 | GO:0001912 | positive regulation of leukocyte mediated cytotoxicity | 7 |
| 104 | GO:0046006 | regulation of activated T cell proliferation | 7 |
| 105 | GO:0045953 | negative regulation of natural killer cell mediated cytotoxicity | 7 |
| 106 | GO:0042116 | macrophage activation | 7 |
| 107 | GO:0045591 | positive regulation of regulatory T cell differentiation | 7 |
| 108 | GO:0032831 | positive regulation of CD4-positive, CD25-positive, alpha-beta regulatory T cell differentiation | 7 |
| 109 | GO:0002501 | peptide antigen assembly with MHC protein complex | 7 |
| 110 | GO:0050821 | protein stabilization | 8 |
| 111 | GO:0006878 | cellular copper ion homeostasis | 8 |
| 112 | GO:0055070 | copper ion homeostasis | 8 |
| 113 | GO:1902904 | negative regulation of supramolecular fiber organization | 9 |
| 114 | GO:1990535 | neuron projection maintenance | 9 |
| 115 | GO:0007008 | outer mitochondrial membrane organization | 9 |
| 116 | GO:0045040 | protein insertion into mitochondrial outer membrane | 9 |
| 117 | GO:0046596 | regulation of viral entry into host cell | 10 |
| 118 | GO:0016045 | detection of bacterium | 10 |
| 119 | GO:0075294 | positive regulation by symbiont of entry into host | 10 |
| 120 | GO:0046598 | positive regulation of viral entry into host cell | 10 |
| 121 | GO:0042270 | protection from natural killer cell mediated cytotoxicity | 10 |
| 122 | GO:0045926 | negative regulation of growth | 11 |

Supplementary Table 3. Pathway analysis related to downregulated genes in MSA-P vs HC in 4 quadrants

| Quadrants | Pathway | Adjusted p-value | Combined score | Genes |
| --- | --- | --- | --- | --- |
| Dorsomedial | Ribosome | 5.09E-47 | 5675.237463 | RPL4;RPL32;RPL31;RPL34;RPLP1;RPL12;RPLP0;RPL36A;RPL9;MRPL10;RPL7;RPS15;RPS14;RPS17;RPS16;RPS18;RPL14;RPLP2;RPL38;RPL37;RPL15;RPS13;RPS12;RPS9;RPL41;RPS8;RPL23;RPL22;RPS6;RPL35A;RPS3A;RPS25;RPS28;RPS29;RPL37A;RPL27;RPS20;FAU;RPL28;RPS21;RPS23 |
|  | Antigen processing and presentation | 3.47E-04 | 137.9719064 | CD74;HSP90AB1;NFYA;HLA-B;CALR;HLA-DPA1;HLA-E |
|  | Mineral absorption | 0.008363 | 89.5812139 | MT2A;FTH1;ATP1A3;MT1X;ATP1B1 |
| Dorsolateral | Ribosome | 3.71E-17 | 2275.59 | RPL5;RPL31;RPL34;RPL22;RPS6;RPS4X;RPS14;RPS28;RPL37A;RPL14;RPLP2;RPS20;RPL15;RPS23 |
| Ventromedial | Ribosome | 1.38E-42 | 8335.644 | RPL32;RPL31;RPL34;RPLP1;RPL36A;RPL9;RPL7;RPL36;RPS3;RPL14;RPLP2;RPL13;RPL38;RPL37;RPL15;RPL19;RPS9;RPL21;RPS8;RPL23;RPL22;RPS6;RPL35A;RPS28;RPS29;RPL37A;RPL24;RPL27;RPS20;RPL26;RPS21;RPS23 |
| Ventrolateral | Ribosome | 5.02E-10 | 1562.734 | RPS14;RPS28;RPS18;RPL34;RPS3A;RPS20;RPL9;RPL7 |
|  | Mineral absorption | 0.03471 | 178.4376 | FTH1;ATP1B1 |

Supplementary Table 4. Gene ontology (Biological Process) clusters for downregulated genes in MSA-P vs PD

|  | id | term | cluster |
| --- | --- | --- | --- |
| 1 | GO:0002181 | cytoplasmic translation | 1 |
| 2 | GO:0043043 | peptide biosynthetic process | 1 |
| 3 | GO:0009059 | macromolecule biosynthetic process | 1 |
| 4 | GO:0006412 | translation | 1 |
| 5 | GO:0010467 | gene expression | 1 |
| 6 | GO:2000059 | negative regulation of ubiquitin-dependent protein catabolic process | 1 |
| 7 | GO:1903051 | negative regulation of proteolysis involved in cellular protein catabolic process | 1 |
| 8 | GO:2000058 | regulation of ubiquitin-dependent protein catabolic process | 1 |
| 9 | GO:0001819 | positive regulation of cytokine production | 1 |
| 10 | GO:0016072 | rRNA metabolic process | 1 |
| 11 | GO:0006417 | regulation of translation | 1 |
| 12 | GO:0022613 | ribonucleoprotein complex biogenesis | 2 |
| 13 | GO:0042254 | ribosome biogenesis | 2 |
| 14 | GO:0042274 | ribosomal small subunit biogenesis | 2 |
| 15 | GO:0030336 | negative regulation of cell migration | 3 |
| 16 | GO:0015914 | phospholipid transport | 3 |
| 17 | GO:0042981 | regulation of apoptotic process | 4 |

Supplementary Table 5. Downregulated genes in MSA-P and PD

| Down regulated genes | DEGs |
| --- | --- |
| MSA-P only | FTH1, ADAM15, APMAP, PIN1, ATP5MC2, TMX3, HPGDS, H1-7, YWHAE, DUX4, TMSB4X, HMGCLL1, GFAP, RPS28, SUPT7L, MYF6, CALM3, KRTDAP, RPS23, TMEM106C, PSMG3, RPL37A, DYNC1H1, CNTLN, MAPKBP1, RPS29, TMEM30A, RPS20, SELENOW, CFL1, RPS6, MFSD6L, CTSZ, CCDC13, MTCH2, DBI, RPS14, CALM2, DHRS1, CYP2W1, MT2A, HSP90AB1, ATP5MG, ZNF28, CTSD, RPL26, PGRMC2, RPL9, TTYH3, S100A6, RPLP2, SERINC1, PDCD4, NFYA, SLFNL1, RPL14, AQP1, RPL4, RPL19, ZNF771, YWHAG, RPL7, RPS12, CCDC137, RPL27, GLUL, PDCD5, COX8A, CD74, C15orf48, AQP4, MAGEA4, TUBB4A, SSB, MEPCE, SPARCL1, RPS18, RPL36A, PMP2, GLRA1, RPL41, CLDN11, RPS3, MBP, ZFP82, FAU, BASP1, RPS17, YWHAQ, KRT28, LDHB, MRFAP1, ITM2B, CALR, RPS8, MARCKSL1, PARP14, CASTOR3, RPL5, SMIM10L1, SARAF, PRKRIP1, IFITM3, RPL36, ZNF346, SLC25A52, DKK3, RPL38, PFDN5, TOMM5, CCDC47, PPP3R1, SLC36A4, NUCKS1, ABHD16A, TMA7, PNO1, QKI, MRPL10, C4B, CD63, RPS27A, RPLP0, PGK1, RPL35A, HLA-DRB1, ADARB1, RPS2, SPANXA1, PABPC1, BTBD6, CTSB, SQSTM1, CRYAB, MT1X, TAGLN, CCDC61, UQCRHL, FCER1G, RPL21, RPL17, RPL8, GPX1, RPS7, HMGB1, RPS13, ITPKC, PPIA, PEA15, UQCRB, MTURN, RPL27A, OBSCN, HLA-A, CEP295NL, RPL12, RPL28, SEPTIN7, BTF3, C1QA, RPL29, TSPAN3, EPC1, RBM3, QDPR, RPS15, WAC, CRHR1, EXOSC6, TUBA1C, ATP6V0C, ACTR2, RPS10, TOMM7, MT1A, MT3, ATP5IF1, MIDN, RPRD1B, RTN3, MT1M, RPS19, TMEM50B, GALNT2, YAF2, SERPINA3, CNBP, CAPNS1, RNASEK, NAP1L1, TUBA3D, EIF3A, STMN3, IFITM2, OPTN, METRNL, RPS27, OAZ1, RAB3C, HLA-E, UBE2V2, RHOA, MAG, EIF1, MEGF8, EIF4G2, SLC7A10, HIPK2, FBF1, RPS16, ANKRD36B, NR1H2, SLC12A9, PLEKHB1, GPX3, BSG, SEC62, BMERB1, MT1E, C11orf58, PSENEN, DDX5, UBXN4, UQCRQ, APOE, SRI, DYNC1LI2, SRD5A1, RPL18A, RPL6, RACK1, LDHA, TRIM51, TUBB2B, EIF1B, CCDC88B, RNF13, CLTA, RPS11, HNRNPA2B1, DSTN, B2M, FCGR3A, PRNP, NDRG1, SRSF5, GNB1, FBRS, H2AJ |
| PD only | INAVA, SEC14L4, ATRX, CLIC3, FRG2C, NFE2L3, RNPEP, DELE1, TXNRD3, TM4SF18, EIF4A2, SLX4, FCGRT, HIRIP3, DAND5, EML6, PPP4C, C5orf38, MAZ, STRN, ECRG4, MYL1, PRRT3, NPNT, MRPS18A, SLURP2, ACTN3, TMEM41B, CIDEA, MBOAT7, C9orf40, DDX4, EBF4, KPNA3, BLOC1S4, CLDN7, HBA2, ADRB3, IMP4, PABIR1, TMIGD2, PPP2R2B, C1QTNF5, TOP1MT, CRYGB, SERP2, F8A2, MIX23, CST2, ETV7, NRSN2, UBQLN2, DICER1, ZNF668, UNC45B, SLC34A2, KRTAP24-1, TECTB, MT1G, TCEAL5, XXYLT1, PKNOX1, PLOD1, ZDHHC21, PTPRD, SELENOH, TMEM80, DTX3, NFKB2, KRTAP9-4, GYPC, ATP6V0B, OSGEP, EGLN1, CPO, RAPGEF5, TNRC18, CREB3L4, KLHL30, STOML1, XAGE3, PRPF8, CCL24, MTO1, ADIRF, FGF12, EMC3, CXCR3, MTPAP, ATP2A1, VN1R1, SRSF11, APBA3, ALDH3B1, STX7, CLEC6A, ACSM2B, ARL8A, CT47B1, KRTAP12-4, COL23A1, NKX1-2, SKP1, LMNTD2, SLC35B2, MED9, MPST, CHD1L, VWF, ERGIC2, SNX33, ELOVL5, SLC22A18AS, TSNAXIP1, DHRS13, PPT1, GABARAPL1, COX6A2, CHRFAM7A, OVCA2, SLC35F4, YJEFN3, RAD51D, WDR26, TAB3, ZFP90, POLR2J3, CNTD1, DOCK6, DEPDC1B, RAB26, SLC34A3, PPP1R3F, TMEM147, RMDN2, AVP, CNTNAP5, ACYP2, DDX27, CHI3L2, IL11RA, NBEA, GAMT, CHST10, ANKRD52, TMPRSS6, AKAP17A, C1QTNF8, GAL3ST2, TMEM210, PIP4K2A, PKD1L2, HOXB1, OR13C4, PABPN1, FBXO21, PTGDR2, OGFOD1, HFE, CLEC3B, RAET1E, DHFR2, NKX2-4, SLC48A1, FOXP4, HMGN1, H2BC4, CLEC19A, PPM1H, CCS, FAM50A, RPRML, COX4I1, POU5F1, KCNJ9, GMFB, USB1, PSPH, NAIF1, NEK10, NPC2, LAG3, PTGFRN, FAM163B, ZBTB22, SSR1, CLEC2L, HNRNPAB, TNC, COX6C, UTF1, XKR6, RHBDD3, CCL18, SAFB2, IFIH1, EVX1, SPRED1, CLDN10, PEX3, SLC6A17, FBXO44, EHD1, IFIT2, SPX, TAF5L, CNN3, GNA11, TSNAX, PUF60, CFHR1, CCNP, INSIG2, CELF4, POPDC3, NAA38, PHLDA2, FOXG1, TYW1, ZNF254, MAPK1IP1L, SLC16A11, LGMN, ITSN1, CYP2C9, C22orf39, CYP2A6, ANKRD18A, FAM181B, PSMD8, SERF2, HOXB3, SNRPN, CSNK1A1, TEPP, TRIM21, CDK4, APLN, CH25H, TMBIM6, HIP1, PTGDS, KLC2, TRPM3, PXN, TBC1D8, IL7, SMPD2, OR4E1, SLC44A5, TRMT112, TTLL9, PCCA, OCIAD2, FCF1, TNFAIP1, EPAS1, RHOU, H2BU1, PMF1, ITGB1, CD200R1L, GPRC5B, TSPAN9, MT1F, RAX2, ASPHD1, MGAT2, COX11, PF4, SLC22A17, ELANE, PTPRA |
| Common | SLU7, STRADA, ALDOA, NPR2, ATP1A3, SPATA2L, LILRB4, ORC4, CMIP, CLU, SNCG, NBPF15, OR10S1, MZT2B, RPL34, PPDPF, PTMA, ZFP30, NDUFB3, PTBP1, HLA-B, ST3GAL2, OOEP, PFN2, UBE2Z, TASOR2, RPLP1, CHP1, RPS3A, TTC33, EEF1G, PEBP1, RPL37, CD40LG, RPL31, PCSK1N, RPL32, RPL22, A2M, APP, RPL24, RPS21, CTBP2, RPS25, COX6A1, TMSB10, SARDH, NPM1, PPP4R3B, RPL15, S100B, OXT, RPL23, SLC22A23, RPS9, RNASE1, ALDH1A1, CIRBP, VIM, SYP, SCD5, PLD3, H3-3B, WBP2, FAM107A, COPS9, CAMK2N1, IGFBP7, RPL30, RPS4Y1, SPARC, NPDC1, RPL13, CKB, LPIN3, RPL11, HLA-DRA |
